# Supplementary material for: Unsupervised transcriptomic analysis of paired pre- and post-treatment specimens reveals divergent chemoimmunomodulatory induction trajectories in breast cancer
Source: bioRxiv. 2026 Sep 28:2026.09.24.754178. Preprint. [Version 1] doi: 10.64898/2026.09.24.754178 (PMC13648446; doi:10.64898/2026.09.24.754178)

bioRxiv preprint doi: https://doi.org/10.64898/2026.09.24.754178; this version posted September 28, 2026. The copyright holder for this preprint (which was not certified by peer review) is the author/funder, who has granted bioRxiv a license to display the preprint in perpetuity. It is made available under a CC-BY 4.0 International license.

SUPPLEMENTAL FIGURES AND FIGURE LEGENDS

Supplemental Figure S1. Differential induction of pyroptotic cell death program. **(A)** Boxplot depicts the relative differential induction (Z-score of average ΔGE values) of pyroptotic cell death program as in **Figure 2F** in Fun-CIM (blue) and Dys-CIM (red) tumors. Statistically significant differences for FDR-adjusted p values are indicated as follows: * ≤ 0.05; ** ≤ 0.01; *** ≤ 0.005; **** ≤ 0.001. **(B-L)** Boxplots display differential gene induction (Δlog2[TPM+1]) in Dys-CIM and Fun-CIM tumors for genes utilized in single-sample gene set analysis, including *AIM2*, *CASP1*, *CASP4*, *CASP5*, *GSDMD*, *GSDME*, *IL1B*, *IL18*, *NLRC4*, *NLRP3*, and *PYCARD.* Stacked bar plots show the proportion of tumors exhibiting induction (green) versus no induction (red) within each trajectory group. p *value*s were computed using the Wilcoxon rank-sum test and Fisher's exact or chi-squared test, as indicated, for comparison of average induction values and proportion of tumors exhibiting induction, respectively.

Supplemental Figure S2. Differential induction of ferroptotic cell death program. **(A)** Boxplot depicts the relative differential induction (Z-score of average ΔGE values) of ferroptotic cell death program as in **Figure 2G** in Fun-CIM (blue) and Dys-CIM (red) tumors. Statistically significant differences for FDR-adjusted p values are indicated as follows: * ≤ 0.05; ** ≤ 0.01; *** ≤ 0.005; **** ≤ 0.001. **(B-F)** Boxplots display differential gene induction (Δlog2[TPM+1]) in Dys-CIM and Fun-CIM tumors for genes utilized in single-sample gene set analysis, including *ACSL4, LPCAT3, PTGS2, SAT1, and TFRC.* Stacked bar plots show the proportion of tumors exhibiting induction (green) versus no induction (red) within each trajectory group. p values were computed using the Wilcoxon rank-sum test and Fisher's exact or chi-squared test for comparison of average induction values and proportion of tumors exhibiting induction, respectively.

Supplemental Figure S3. Differential induction of PANoptotic cell death program. **(A)** Boxplot depicts the relative differential induction (Z-score of average ΔGE values) of PANoptotic cell death program as in **Figure 2H** in Fun-CIM (blue) and Dys-CIM (red)

bioRxiv preprint doi: https://doi.org/10.64898/2026.09.24.754178; this version posted September 28, 2026. The copyright holder for this preprint (which was not certified by peer review) is the author/funder, who has granted bioRxiv a license to display the preprint in perpetuity. It is made available under a CC-BY 4.0 International license.

tumors. Statistically significant differences for FDR-adjusted p values are indicated as follows: * ≤ 0.05; ** ≤ 0.01; *** ≤ 0.005; **** ≤ 0.001. **(B-I)** Boxplots display differential gene induction (Δlog2[TPM+1]) in Dys-CIM and Fun-CIM tumors for genes utilized in single-sample gene set analysis, including *AIM2, CASP8, FADD, IRF1, RIPK1, RIPK3, PYCARD, and ZBP1.* Stacked bar plots show the proportion of tumors exhibiting induction (green) versus no induction (red) within each trajectory group. p values were computed using the Wilcoxon rank-sum test and Fisher's exact or chi-squared test for comparison of average induction values and proportion of tumors exhibiting induction, respectively.

Supplemental Figure S4. Differential induction of necroptotic cell death program. **(A)** Boxplot depicts the relative differential induction (Z-score of average ΔGE values) of necroptotic cell death program as in **Figure 2I** in Fun-CIM (blue) and Dys-CIM (red) tumors. Statistically significant differences for FDR-adjusted p values are indicated as follows: * ≤ 0.05; ** ≤ 0.01; *** ≤ 0.005; **** ≤ 0.001. **(B-F)** Boxplots display differential gene induction (Δlog2[TPM+1]) in Dys-CIM and Fun-CIM tumors for genes utilized in single-sample geneset analysis, including *MLKL*, *RIPK1*, *RIPK3*, *TICAM1*, and *ZBP1.* Stacked bar plots show the proportion of tumors exhibiting induction (green) versus no induction (red) within each trajectory group. p values were computed using the Wilcoxon rank-sum test and Fisher's exact or chi-squared test for comparison of average induction values and proportion of tumors exhibiting induction, respectively.

Supplemental Figure S5. Differential induction of apoptotic cell death program. **(A)** Boxplot depicts the relative differential induction (Z-score of average ΔGE values) of apoptotic cell death program as in **Figure 2J** in Fun-CIM (blue) and Dys-CIM (red) tumors. Statistically significant differences for FDR-adjusted p values are indicated as follows: * ≤ 0.05; ** ≤ 0.01; *** ≤ 0.005; **** ≤ 0.001. **(B-U)** Boxplots display differential gene induction (Δlog2[TPM+1]) in Dys-CIM and Fun-CIM tumors for genes utilized in single-sample geneset analysis, including *APAF1, BAD, BAK1, BAX, BBC3, BCL2L11, BID, BMF, CASP3, CASP7, CASP8, CASP9, CYCS, DIABLO, FADD, FAS, HRK, PMAIP1, TNFRSF10B, and TP53AIP1.* Stacked bar plots show the proportion of tumors exhibiting induction (green) versus no induction (red) within each trajectory group. p values were

bioRxiv preprint doi: https://doi.org/10.64898/2026.09.24.754178; this version posted September 28, 2026. The copyright holder for this preprint (which was not certified by peer review) is the author/funder, who has granted bioRxiv a license to display the preprint in perpetuity. It is made available under a CC-BY 4.0 International license.

computed using the Wilcoxon rank-sum test and using Fisher's exact or chi-squared test, as indicated, for comparison of average induction values and proportion of tumors exhibiting induction, respectively.

Supplemental Figure S6. Differential induction of ICD genes across CIM trajectories. (A-H) Boxplots display differential gene induction (Δlog2[TPM+1]) in Dys-CIM and Fun-CIM tumors *EIF2AK3, EIF2A, ATF4, CALR, PDIA3, HMGB1, and ANXA1*. Stacked bar plots show the proportion of tumors exhibiting induction (green) versus no induction (red) within each trajectory group. Boxed legend applies to adjacent stacked bar plots indicating percentage of samples with positive induction (ΔGE > 0) or negative/no induction (ΔGE ≤ 0). p values were computed using the Wilcoxon rank-sum test and using Fisher's exact or chi-squared test, as indicated, for comparison of average induction values and proportion of tumors exhibiting induction, respectively.

Supplemental Figure S7. Fun-CIM tumors have greater induction of CIM sensors. (A-H) Boxplots display differential gene induction (Δlog2[TPM+1]) in Dys-CIM (blue) and Fun-CIM (red) tumors for key innate immune sensing and interferon signaling genes, including *IFI16*, *TLR9*, *TLR3*, *TLR7*, *TLR8*, *P2RX7*, *FPR1*, *TLR4*, *IFNAR1*, *IFNAR2*, *IFNGR1*, and *STING1*. Stacked bar plots show the proportion of tumors exhibiting induction (green) versus no induction (red) within each trajectory group. Boxed legend applies to stacked barplots indicating the percentage of samples with positive induction (ΔGE > 0) or negative/no induction (ΔGE ≤ 0). p values were computed using the Wilcoxon rank-sum test and using Fisher's exact test for comparison of average induction values and proportion of tumors exhibiting induction, respectively.

Supplemental Figure S8. Differential induction of antigen-presentation genes and immune checkpoint-related genes across CIM trajectories. (A–L) Boxplots show differential gene induction (Δlog2[TPM+1]) between pre- and post-chemotherapy samples in Dys-CIM and Fun-CIM tumors for *NFKB1* **(A)**, Avg_MHC1 (average expression of *HLA-A, HLA-B,* and *HLA-C*) **(B)**, *PDCD1* **(C)**, *HHLA2* **(D)**, *CD274* (PD-L1) **(E)**, *CTLA4* **(F)**, *LAG3* **(G)**, *VTCN1* **(H)**, *BTLA* **(I)**, *HAVCR2* **(J)**, *VSIR* **(K)**, and *TIGIT* **(L)**.

bioRxiv preprint doi: https://doi.org/10.64898/2026.09.24.754178; this version posted September 28, 2026. The copyright holder for this preprint (which was not certified by peer review) is the author/funder, who has granted bioRxiv a license to display the preprint in perpetuity. It is made available under a CC-BY 4.0 International license.

Adjacent stacked bar plots show the proportion of tumors exhibiting positive induction (ΔGE > 0; green) versus no or negative induction (ΔGE ≤ 0; red) within each CIM trajectory. Fun-CIM tumors exhibited greater induction of multiple antigen-presentation and immune checkpoint-related genes compared with Dys-CIM tumors. p values above boxplots indicate comparisons of ΔGE between CIM trajectories using the Wilcoxon rank-sum test, whereas p values above stacked bar plots indicate comparisons of the proportions of tumors exhibiting positive versus no/negative induction using Fisher's exact or chi-squared test, as indicated.

Supplemental Figure S9. Biopsy-to-surgery changes in CD8$^+$ T-cell abundance across CIM trajectories in NKI/SMC cohorts. Paired comparisons of immune-cell abundance between pre-treatment biopsy and post-treatment surgical specimens in Fun-CIM tumors (n = 25) and Dys-CIM tumors (n = 11). **(A)** CD8$^+$ T-cell abundance estimated using quanTIseq, MCP-counter, ConsensusTME, xCell, CIBERSORTx, EPIC, and TIMER. **(B)** NK-cell abundance estimated using ConsensusTME, MCP-counter, quanTIseq, CIBERSORTx, and EPIC. Each dot represents an individual tumor sample, with lines connecting matched biopsy and surgical specimens. Dys-CIM tumors exhibited significant reductions in CD8$^+$ T-cell abundance following chemotherapy across quanTIseq, MCP-counter, ConsensusTME, xCell, and EPIC methods. NK-cell abundance was also significantly reduced in Dys-CIM tumors by ConsensusTME and MCP-counter, whereas no significant overall reduction was observed in Fun-CIM tumors, with a subset of patients showing increased in CD8$^+$ T-cell and NK-cell abundance following treatment. Displayed p *value*s were calculated using two-sided paired Wilcoxon signed-rank tests.

Supplemental Figure S10. Validation of enhanced CIM sensor induction in Fun-CIM tumors in the NEO cohort. (A–L) Boxplots show differential gene induction (Δlog2[TPM+1]) between pre- and post-chemotherapy samples in Dys-CIM (blue) and Fun-CIM (red) tumors for key innate immune sensing and interferon signaling genes, including *STING1* (A), *IFNGR1* (B), *IFNAR2* (C), *IFNAR1* (D), *IFI16* (E), *FPR1* (F), *TLR9* (G), *TLR8* (H), *TLR7* (I), *TLR4* (J), *TLR3* (K), and *P2RX7* (L). Adjacent stacked bar plots show the proportion of tumors exhibiting induction (green) versus no induction (red) within

bioRxiv preprint doi: https://doi.org/10.64898/2026.09.24.754178; this version posted September 28, 2026. The copyright holder for this preprint (which was not certified by peer review) is the author/funder, who has granted bioRxiv a license to display the preprint in perpetuity. It is made available under a CC-BY 4.0 International license.

each trajectory group. Boxed legend applies to stacked barplots indicating percentage of samples with positive induction (ΔGE > 0) or negative/no induction (ΔGE ≤ 0) p values were computed using the Wilcoxon rank-sum test and Fisher's exact test for comparison of average induction values and proportion of tumors exhibiting induction, respectively.

Supplemental Figure S11. Induction of inflammatory signaling, antigen-presentation, and immune checkpoint-related genes across CIM trajectories in the NEO cohort. (A–L) Boxplots show differential gene induction (Δlog2[TPM+1]) between pre- and post-chemotherapy samples in Dys-CIM (blue) and Fun-CIM (red) tumors for *NFKB1* (A), Avg_MHC1 (average expression of *HLA-A, HLA-B,* and *HLA-C*) (B), *VSIR* (C), *HHLA2* (D), *TIGIT* (E), *VTCN1* (F), *PDCD1* (G), *CD274* (PD-L1) (H), *CTLA4* (I), *LAG3* (J), *BTLA* (K), and *HAVCR2* (L). Adjacent stacked bar plots show the proportion of tumors exhibiting positive induction (ΔGE > 0; green) versus no or negative induction (ΔGE ≤ 0; red) within each CIM trajectory. Fun-CIM tumors exhibited significantly greater induction of *NFKB1* and *VSIR*, with a significantly higher proportion of Fun-CIM tumors exhibiting positive induction of both genes. p values above boxplots indicate comparisons of ΔGE between CIM trajectories using the Wilcoxon rank-sum test, whereas p values above stacked bar plots indicate comparisons of the proportions of tumors exhibiting positive versus no/negative induction using Fisher's exact test.

Supplemental Figure S12. PAM50 subtyping and deconvolution-based immune profiling by CIMIC derived trajectories in the NEO cohort. Stacked bar plots showing the distributions of PAM50 molecular subtypes in Dys-CIM and Fun-CIM tumors at pretreatment biopsy **(A)** and post-treatment surgery **(B)**. Displayed *p*-values were calculated using Fisher's exact test. **(C)** Heatmap summarizing paired biopsy-to-surgery changes in immune-cell abundance and immune-related scores estimated using eight deconvolution methods. Changes were calculated as surgery minus biopsy and standardized as Z scores. Rows represent immune-cell types or immune scores, and columns represent the mean Z-scored change within each CIM trajectory; red and blue indicate relative increases and decreases, respectively. **(D)** Paired biopsy-to-surgery changes in ConsensusTME cytotoxicity scores and $CD8^{+}$ T-cell abundance estimated

bioRxiv preprint doi: https://doi.org/10.64898/2026.09.24.754178; this version posted September 28, 2026. The copyright holder for this preprint (which was not certified by peer review) is the author/funder, who has granted bioRxiv a license to display the preprint in perpetuity. It is made available under a CC-BY 4.0 International license.

using CIBERSORTx, ConsensusTME, EPIC, and MCP-counter. **(E)** Paired changes in NK-cell abundance estimated using quanTIseq, EPIC, MCP-counter, ConsensusTME, and xCell. Each dot represents an individual tumor sample, with lines connecting matched biopsy and surgical specimens. Displayed *p*-values for paired comparisons were calculated using two-sided Wilcoxon signed-rank tests.

Supplemental Figure S13. Fun-CIM and Dys-CIM TNBC cell lines exhibit divergent transcriptional programs induced by chemotherapy. Top 15 enriched Gene Ontology (GO) biological processes among genes preferentially induced in Fun-CIM (A) and Dys-CIM (B) cell lines following 48 hours of epirubicin treatment. Bar length represents the number of genes overlapping each GO biological process, and color indicates the FDR-adjusted p value.

Supplemental Figure S14. Induction of immune checkpoints in TNBC cell lines. (A-H) (A–M) Boxplots show differential gene induction (Δlog2[TPM+1]) following epirubicin treatment in Dys-CIM (blue) and Fun-CIM (red) cell lines for Avg_MHC1 (average expression of *HLA-A, HLA-B,* and *HLA-C*) **(A)**, *PDCD1* **(B)**, *VSIR* **(C)**, *LGALS9* **(D)**, *HHLA2* **(E)**, *CD274* (PD-L1) **(F)**, *TIGIT* **(G)**, *LAG3* **(H)**, *PDCD1LG2* (PD-L2) **(I)**, *BTLA* **(J)**, *HAVCR2* **(K)**, *CTLA4* **(L)**, and *VTCN1* **(M)**. Adjacent stacked bar plots show the proportion of samples exhibiting positive induction (ΔGE > 0; green) versus no or negative induction (ΔGE ≤ 0; red) within each CIM trajectory. Fun-CIM cell lines exhibited greater induction of several immune checkpoint-related genes, including *VSIR, LGALS9, HHLA2, TIGIT,* and *VTCN1*. p values above boxplots indicate comparisons of ΔGE between CIM trajectories using the Wilcoxon rank-sum test, whereas p values above stacked bar plots indicate comparisons of the proportions of samples exhibiting positive versus no/negative induction using Fisher's exact or chi-squared test, as indicated.

Supplemental Figure S15. Fun-CIM trajectories exhibit greater induction of viral mimicry programs and cytosolic nucleic acid sensors in response to chemotherapy across tumors and cell line datasets. (A–F) Relative differential induction of a 47-gene viral mimicry signature in Fun-CIM and Dys-CIM tumors from the

bioRxiv preprint doi: https://doi.org/10.64898/2026.09.24.754178; this version posted September 28, 2026. The copyright holder for this preprint (which was not certified by peer review) is the author/funder, who has granted bioRxiv a license to display the preprint in perpetuity. It is made available under a CC-BY 4.0 International license.

NKI/SMC and NEO cohorts and in TNBC cell lines. (A) Box plot depicts the relative differential induction (Z-score of average ΔGE values) for 47-gene viral mimicry signature, and (B) Heatmap depicts the relative differential induction of genes used for the viral mimicry signature in Fun-CIM and Dys-CIM tumors in the NKI/SMC cohorts using Z-score of average ΔGE values. (C–D) Corresponding analysis of the viral mimicry signature in the NEO cohort, showing the overall program induction (C) and individual signature genes (D). (E-F) Viral mimicry induction in TNBC cell lines following epirubicin treatment, showing the overall program induction (E) and individual signature genes (F). (G–L) Boxplots show differential gene induction (Δlog2[TPM+1]) in Dys-CIM and Fun-CIM cell lines for key cytosolic nucleic acid sensing genes, including *IFIH1* (G), *CGAS* (H), *STING1* (I), *AIM2* (J), *DHX58* (K), and *DDX58* (RIG-I) (L). Adjacent stacked bar plots show the proportion of samples exhibiting positive induction (ΔGE > 0; green) versus no or negative induction (ΔGE ≤ 0; red) within each CIM trajectory. Fun-CIM cell lines exhibited greater induction of multiple cytosolic nucleic acid sensors, including *IFIH1, STING1, AIM2,* and *DHX58*. p values above boxplots in (G–L) indicate comparisons of ΔGE between CIM trajectories using the Wilcoxon rank-sum test, whereas p values above stacked bar plots indicate comparisons of the proportions of samples exhibiting positive versus no/negative induction using Fisher's exact or chi-squared test, as indicated. FDR-adjusted p values for viral mimicry program and gene-level comparisons in (A–F) are indicated as follows: * ≤ 0.05; ** ≤ 0.01; *** ≤ 0.005; **** ≤ 0.001.

Supplemental Figure S16. Conserved Fun-CIM and Dys-CIM transcriptional programs across patient cohorts and TNBC cell lines. (A, C) Stouffer conservation analysis of directionally concordant genes across NKI/SMC, NEO, and TNBC cell lines identified 551 conserved Fun-CIM (A) and 6 Dys-CIM (C) genes. (B, D) Gene Ontology overrepresentation analysis showed enrichment of immune and inflammatory programs in Fun-CIM (B) and protein maintenance and metabolic programs in Dys-CIM (D). (E–F) Conservation analysis of CIM-related (E) and regulated cell-death (F) pathways demonstrated conserved induction of immunostimulatory and inflammatory cell-death programs in Fun-CIM across all three datasets.

bioRxiv preprint doi: https://doi.org/10.64898/2026.09.24.754178; this version posted September 28, 2026. The copyright holder for this preprint (which was not certified by peer review) is the author/funder, who has granted bioRxiv a license to display the preprint in perpetuity. It is made available under a CC-BY 4.0 International license.

Supplemental Figure S17. Association of conserved Fun-CIM and Dys-CIM transcriptional remodeling with recurrence-free survival in the NKI cohort. (A–B) Directional distribution of gene-level associations with recurrence-free survival (RFS) for conserved Dys-CIM and Fun-CIM gene sets identified by the NKI/SMC + TNBC cell line (A) and NKI/SMC + NEO + TNBC cell line (B) meta-analyses. For each gene, change was calculated as surgery minus biopsy $\Delta\log_2$(TPM+1) expression and standardized within gene. Genes were evaluated individually using univariable Cox proportional-hazards models and classified as beneficial ($P < 0.05$, HR < 1), detrimental ($P < 0.05$, HR > 1), or not significant (NS). (C–D) Associations between longitudinal changes in ssGSEA scores for the corresponding conserved Fun-CIM and Dys-CIM signatures and RFS. Hazard ratios represent the association per 1-SD increase in ΔssGSEA score. Increased Fun-CIM scores were associated with improved RFS, whereas increased Dys-CIM scores were associated with poorer RFS. HR, hazard ratio; CI, confidence interval; ssGSEA, single-sample gene set enrichment analysis.

Supplemental Figure S18. Dys-CIM tumors in the NKI/SMC cohorts exhibit baseline overrepresentation of immune-related transcriptional programs and greater immune enrichment than Fun-CIM tumors. (A–B) Top 15 enriched Gene Ontology Biological Process (GO BP) terms among genes with higher baseline expression in Dys-CIM (A) or Fun-CIM (B) tumors from the combined NKI/SMC cohort. Baseline differentially expressed genes were identified using limma-trend with Benjamini–Hochberg-adjusted p ≤ 0.05 and $|\log_2$ fold change$| \geq \log_2(1.25)$. (C) Baseline overall immune enrichment in Fun-CIM (n = 25) and Dys-CIM (n = 11) tumors, estimated using the xCell and ESTIMATE immune scores. (D–E) Baseline abundance of $CD8^+$ T cells (D) and NK cells (E) estimated using the indicated immune-deconvolution methods. Dys-CIM tumors exhibited higher baseline $CD8^+$ T-cell abundance across multiple methods, whereas differences in NK-cell abundance were less consistent. Displayed p *value*s were calculated using two-sided Wilcoxon rank-sum test.

Supplemental Figure S19. Dys-CIM tumors exhibit higher baseline MHC-I and immune checkpoint-related gene expression than Fun-CIM tumors. (A–M) Boxplots

bioRxiv preprint doi: https://doi.org/10.64898/2026.09.24.754178; this version posted September 28, 2026. The copyright holder for this preprint (which was not certified by peer review) is the author/funder, who has granted bioRxiv a license to display the preprint in perpetuity. It is made available under a CC-BY 4.0 International license.

show baseline $\log_2$(TPM + 1) expression of Avg_MHC1 (mean expression of *HLA-A, HLA-B,* and *HLA-C*) (A) and immune checkpoint-related genes, including *PDCD1* (B), *VSIR* (C), *LGALS9* (D), *HHLA2* (E), *CD274* (PD-L1) (F), *TIGIT* (G), *LAG3* (H), *PDCD1LG2* (PD-L2) (I), *BTLA* (J), *HAVCR2* (K), *CTLA4* (L), and *VTCN1* (M) in Dys-CIM (n = 11) and Fun-CIM (n = 25) tumors from the NKI/SMC cohorts. Dys-CIM tumors exhibited significantly higher baseline Avg_MHC1 and expression of multiple immune checkpoint-related genes, including *PDCD1, LGALS9, CD274, TIGIT, LAG3, PDCD1LG2,* and *CTLA4*. Displayed p values are Benjamini–Hochberg FDR-adjusted and were estimated using limma with robust, trend-aware empirical Bayes moderation.

bioRxiv preprint doi: https://doi.org/10.64898/2026.09.24.754178; this version posted September 28, 2026. The copyright holder for this preprint (which was not certified by peer review) is the author/funder, who has granted bioRxiv a license to display the preprint in perpetuity. It is made available under a CC-BY 4.0 International license.

1

Unsupervised transcriptomic analysis of pre- and post-treatment specimens identifies chemoimmunomodulatory induction trajectories in breast cancer

Supplemental Figures

Supplemental Figure S1

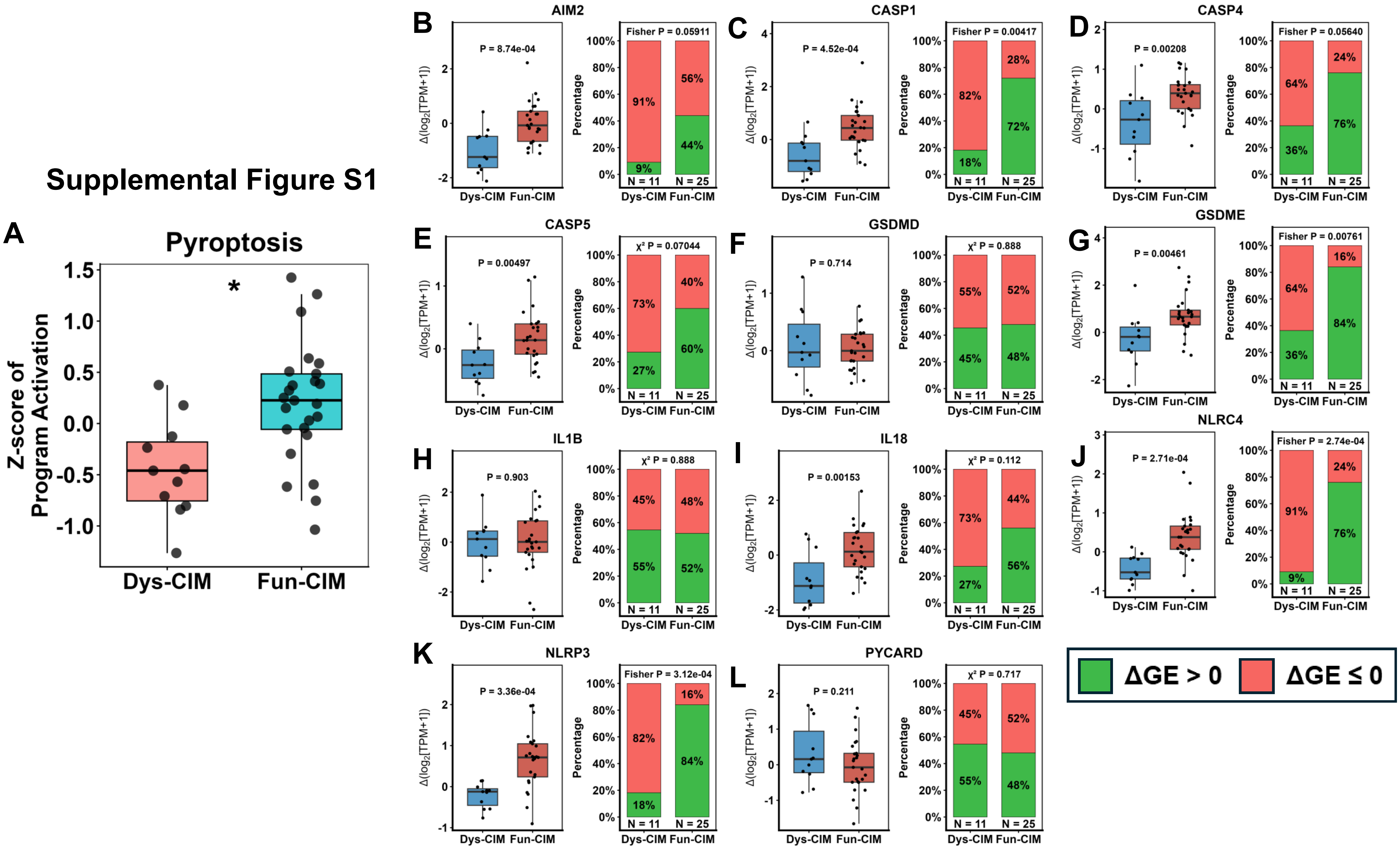

Supplemental Figure S2

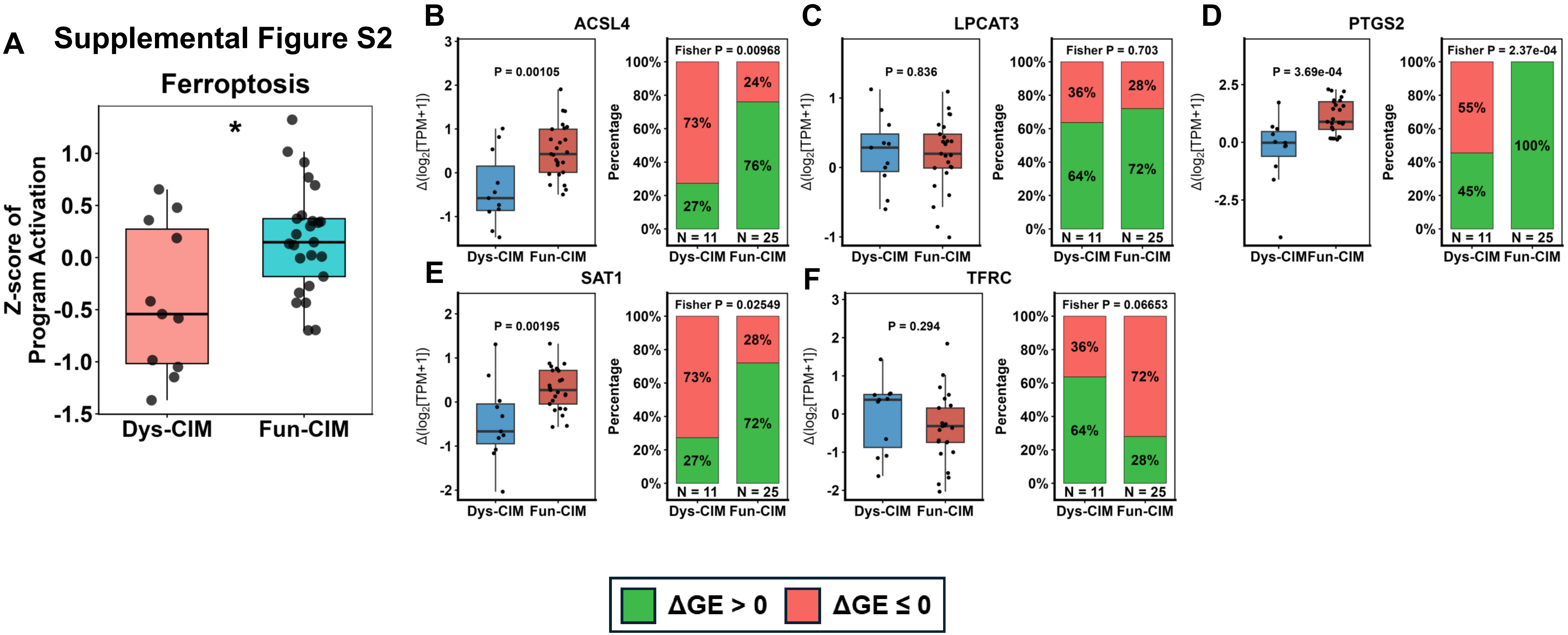

Supplemental Figure S3

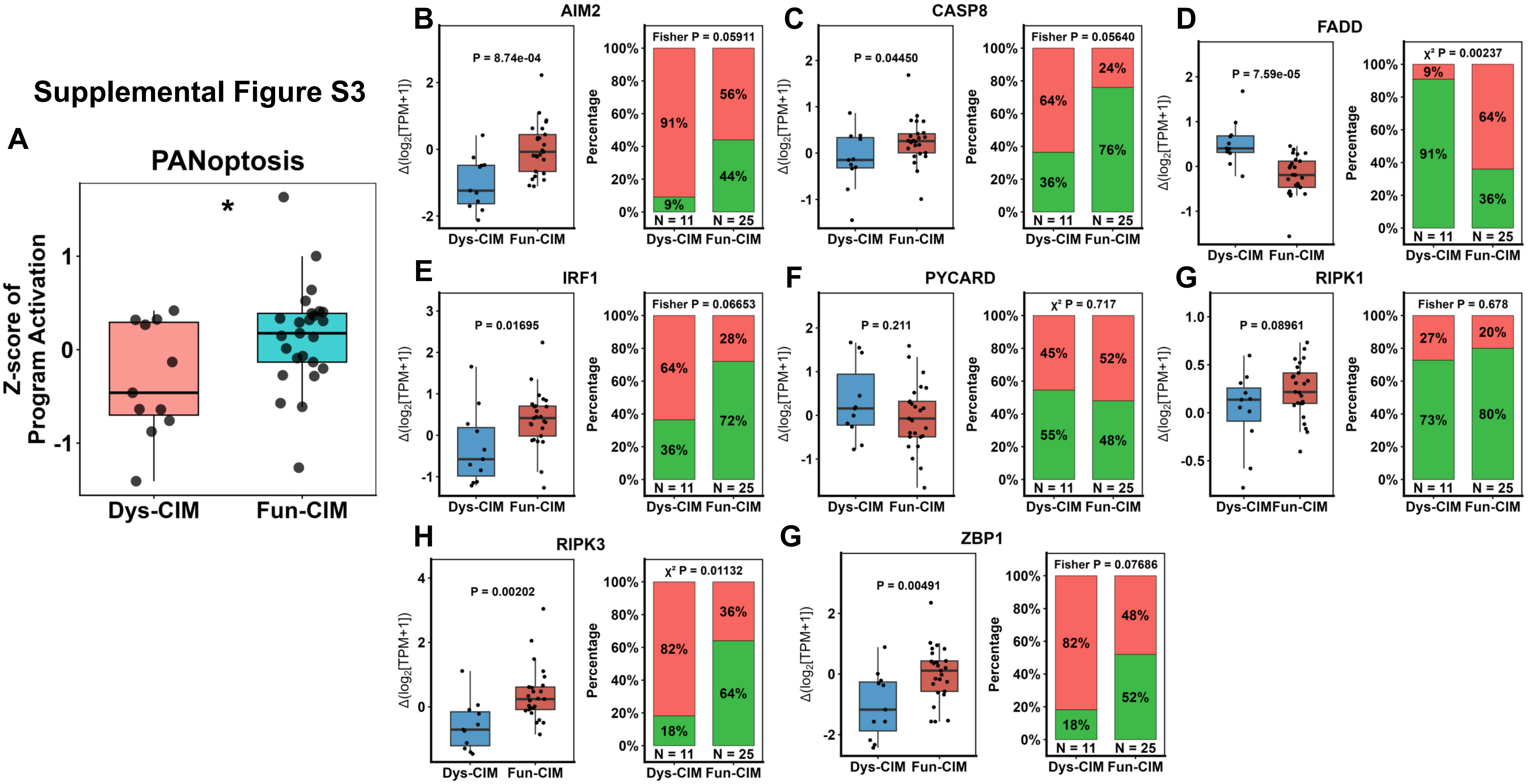

Supplemental Figure S4

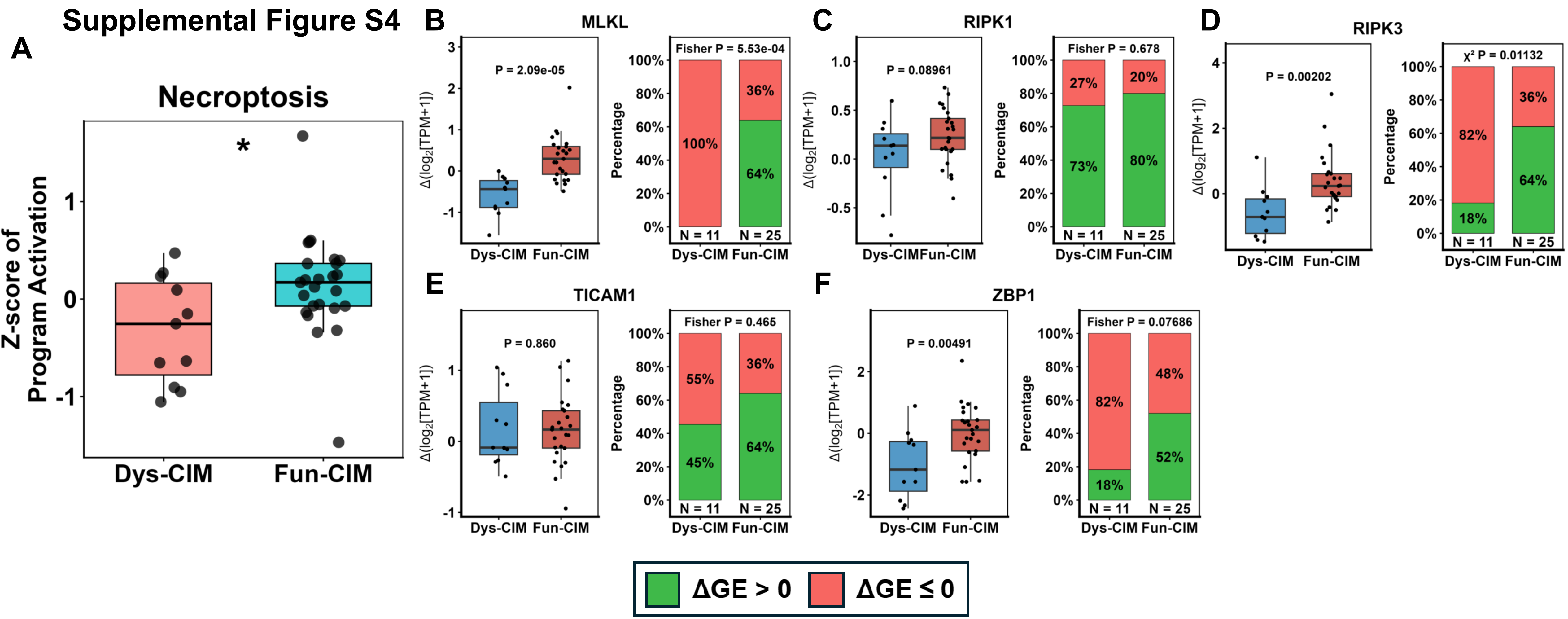

Supplemental Figure S5

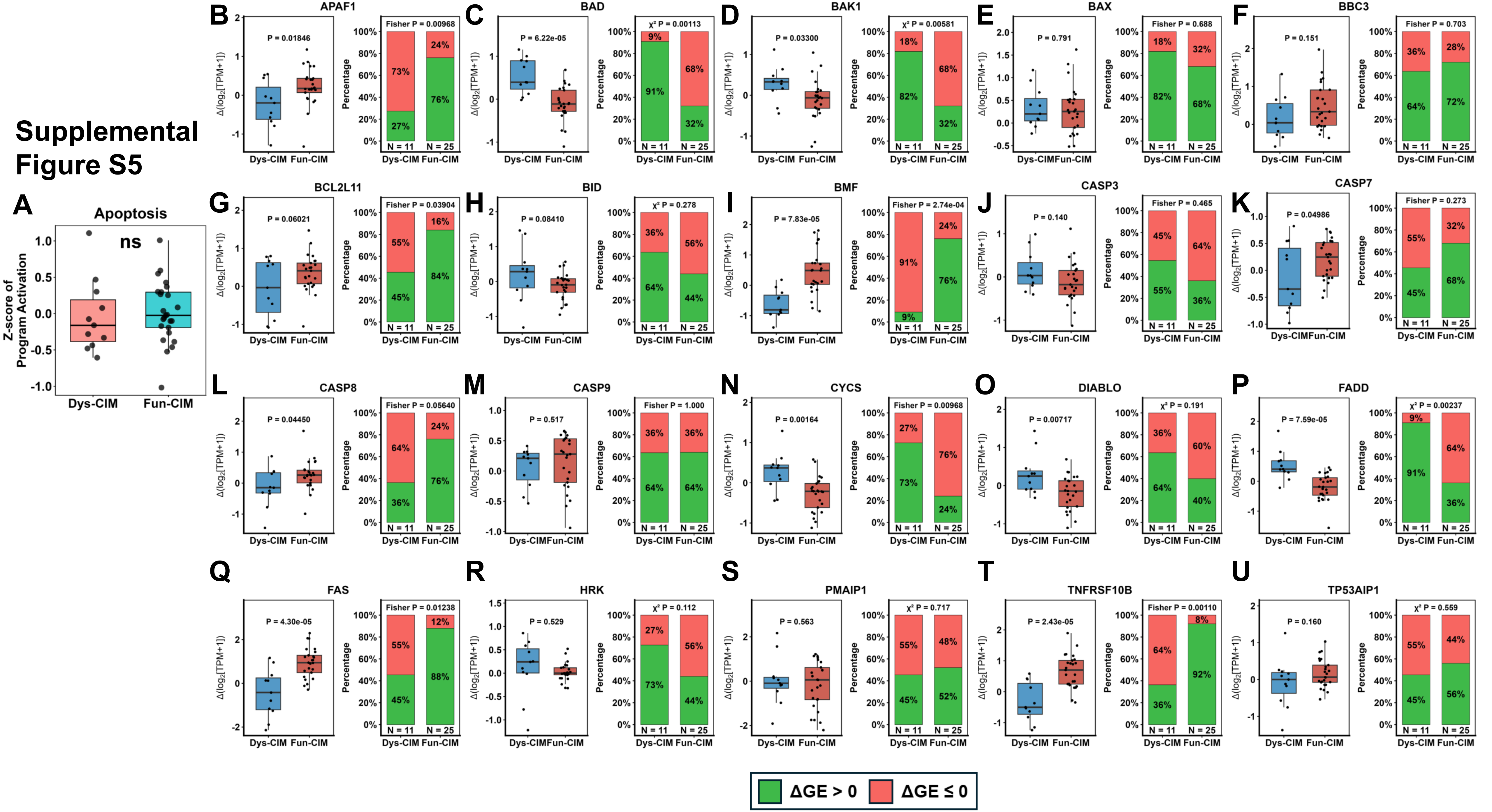

Supplemental Figure S6

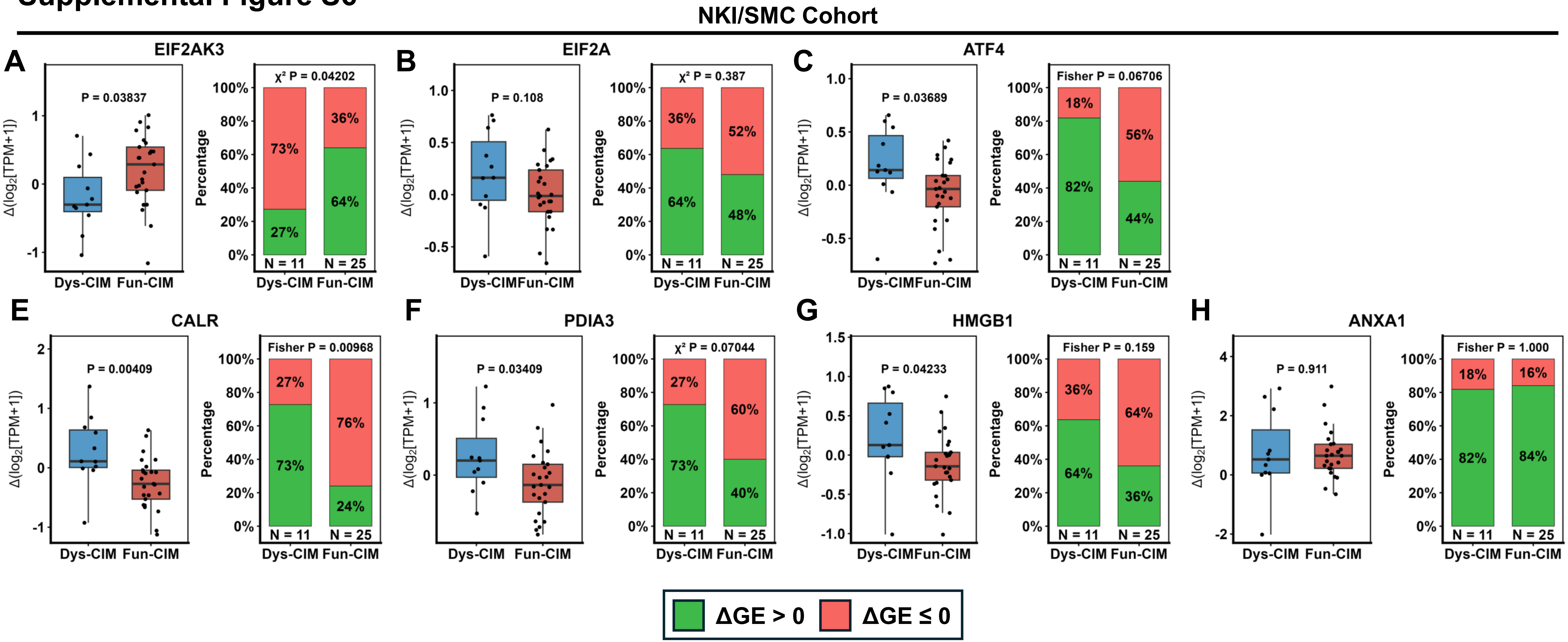

Supplemental Figure S7

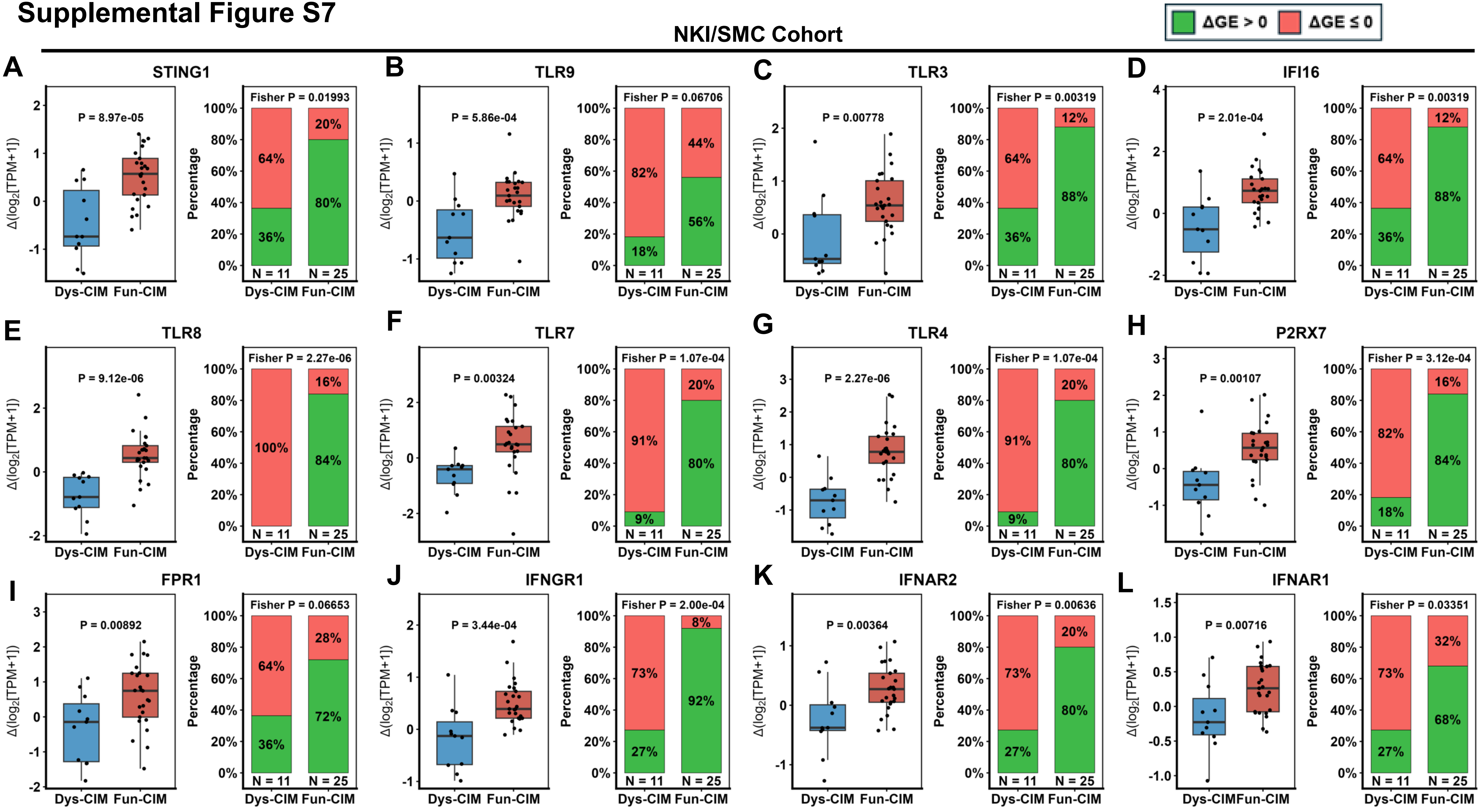

Supplemental Figure S8

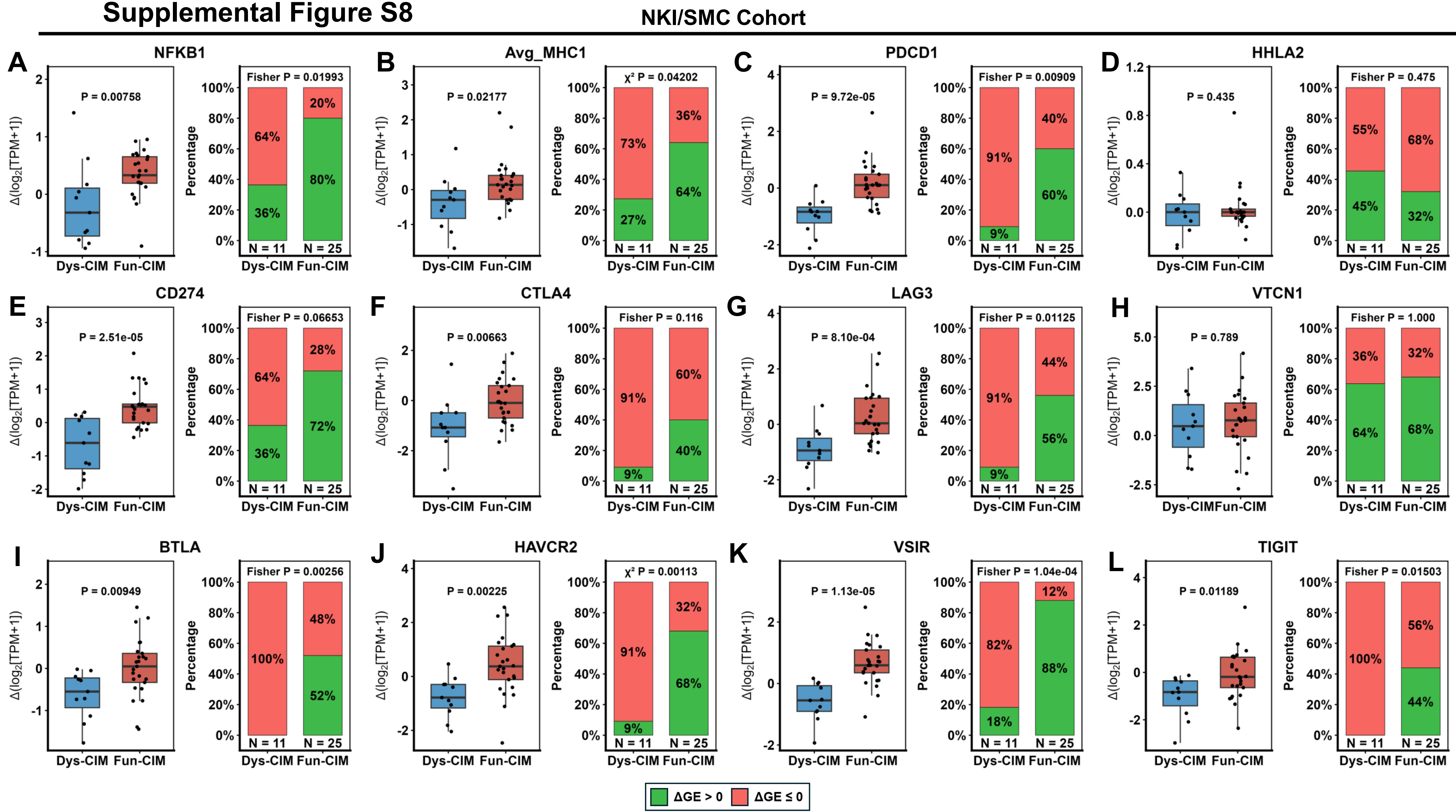

Supplemental Figure S9

NKI/SMC Cohort

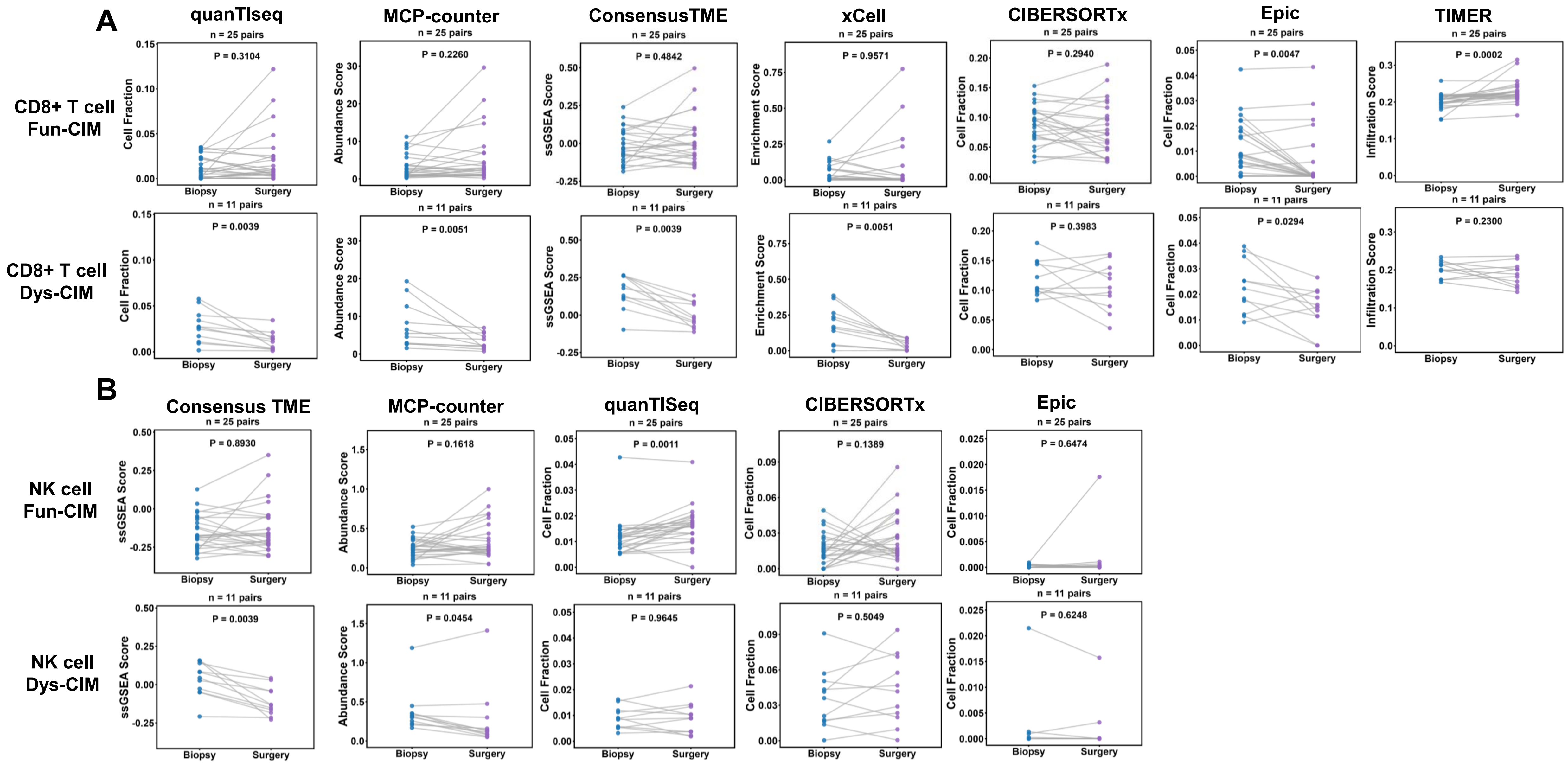

Supplemental Figure S10 NEO External Validation

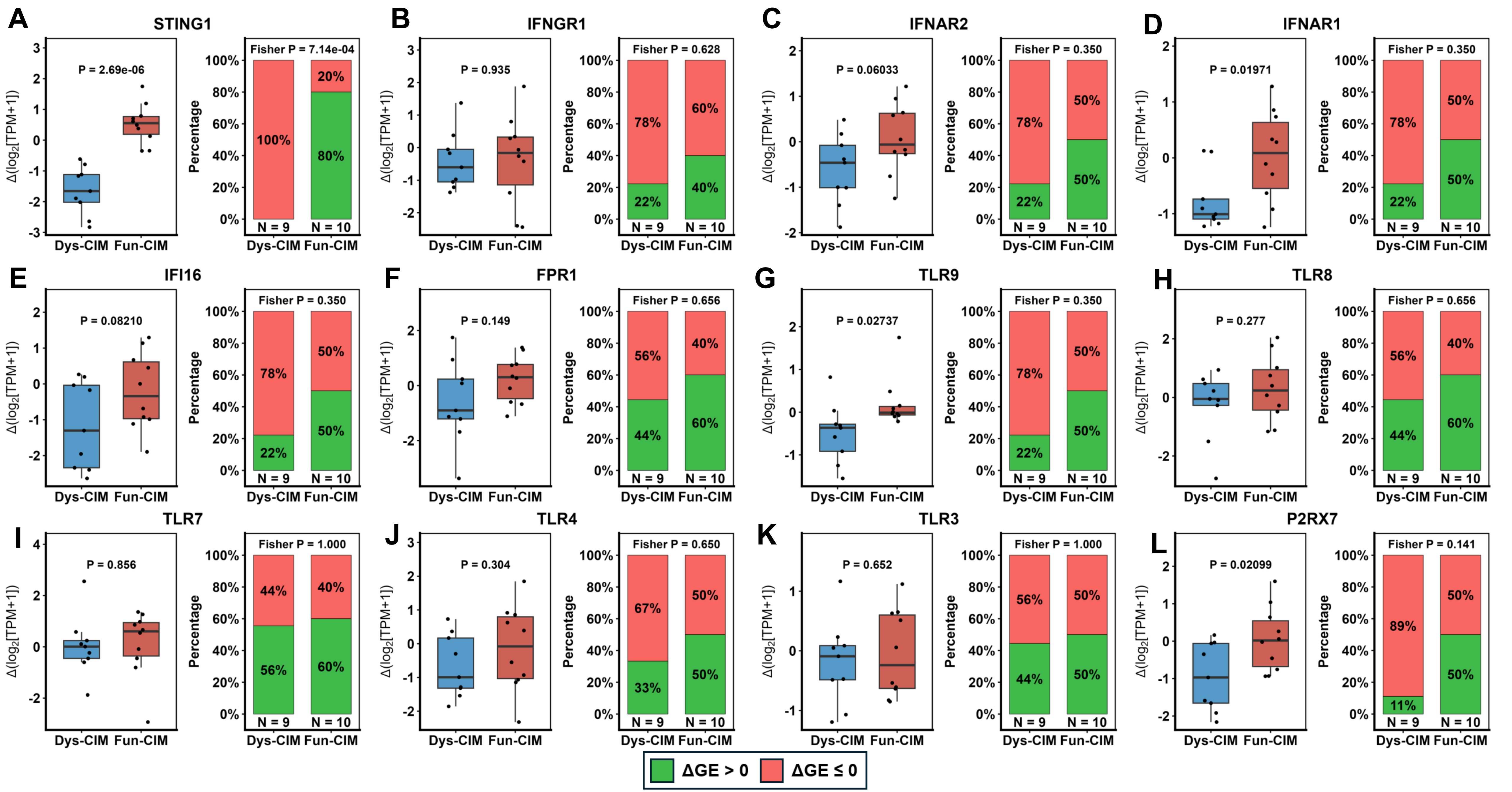

Supplemental Figure S11

NEO External Validation

ΔGE > 0 | ΔGE ≤ 0

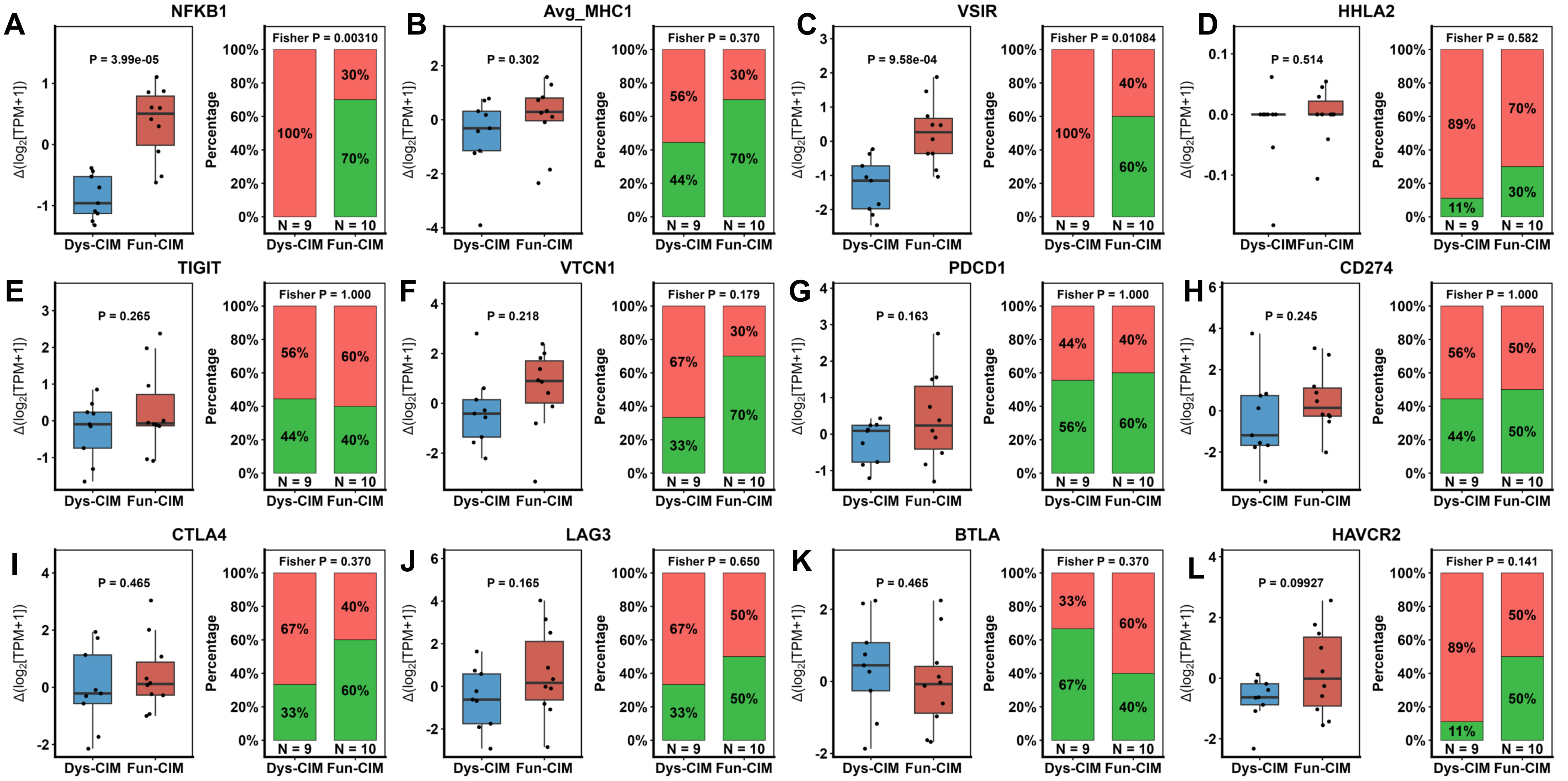

Supplemental Figure S12

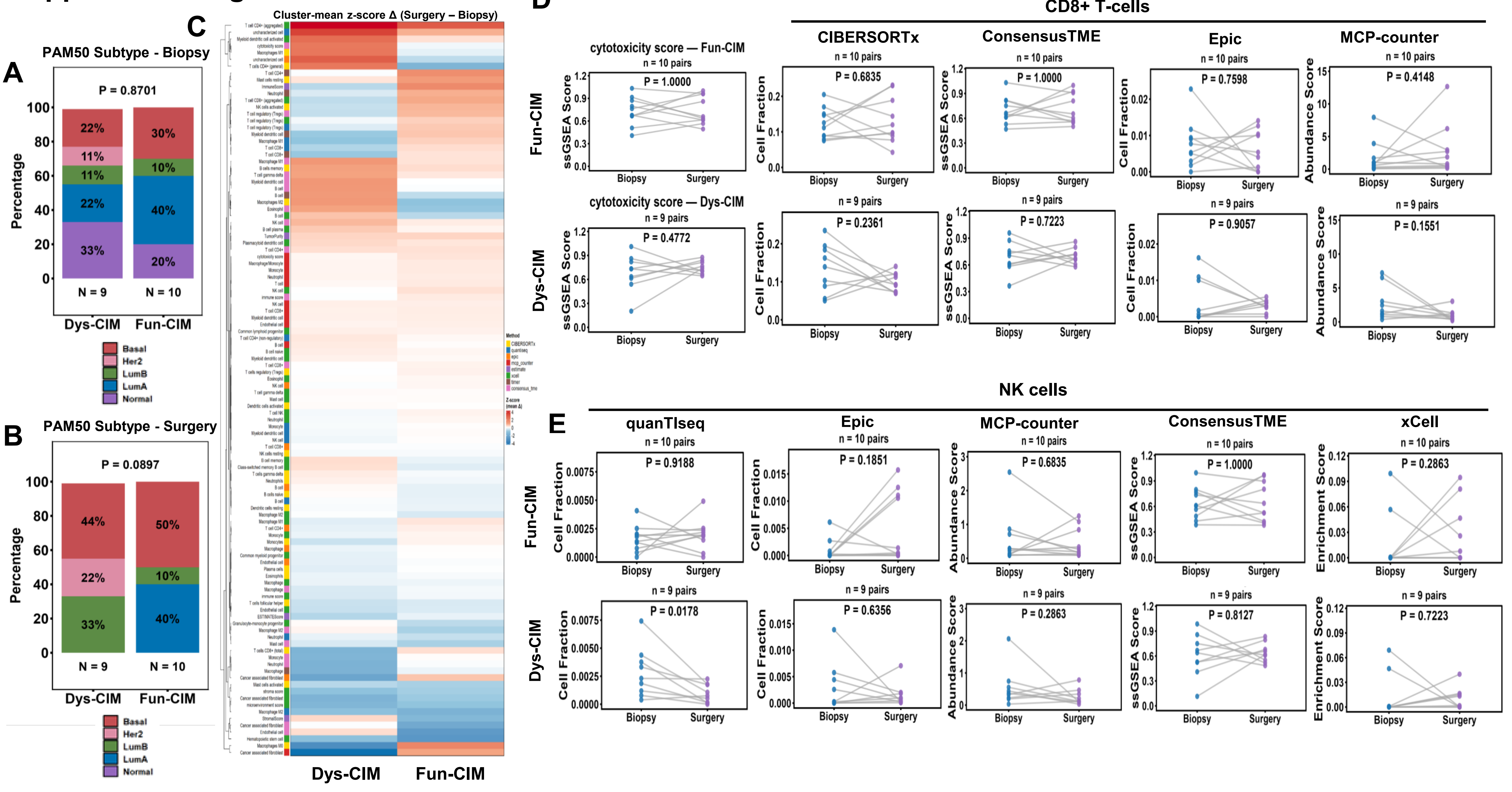

Supplemental Figure S13

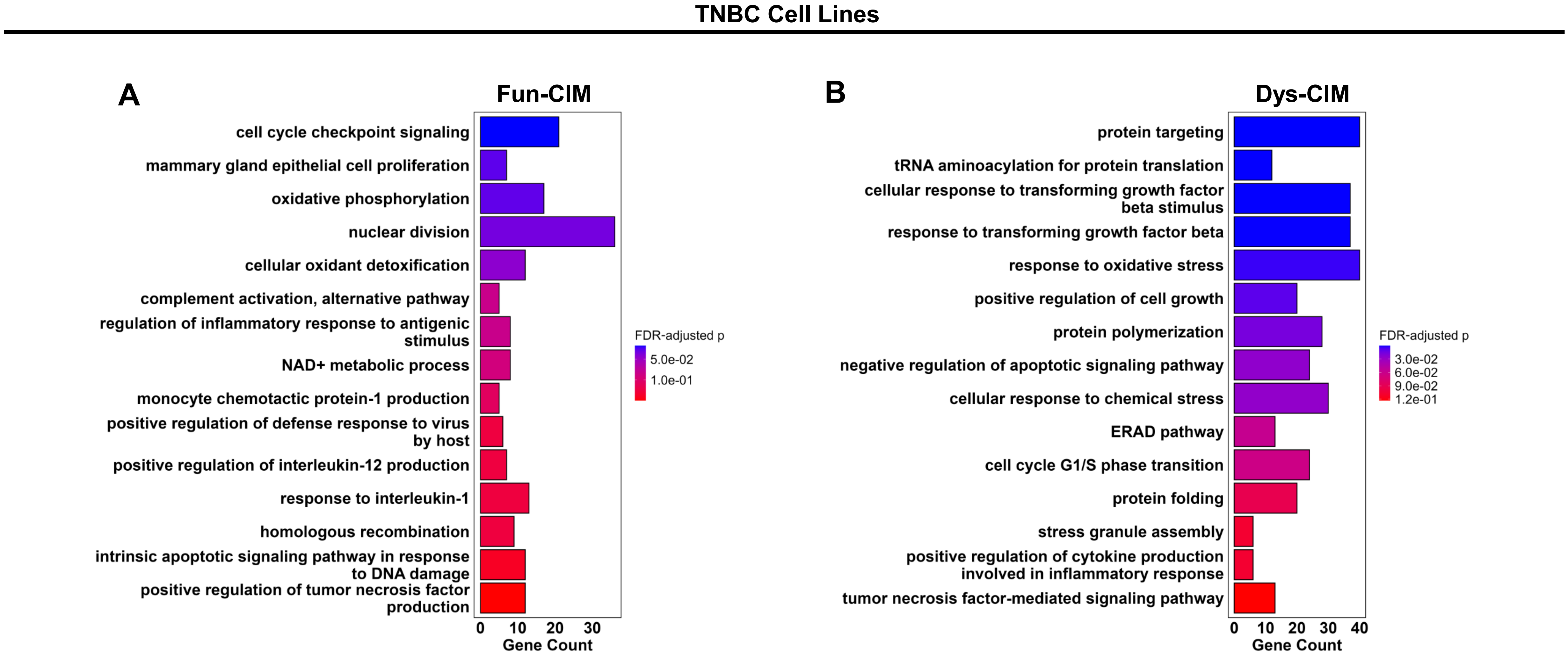

Supplemental Figure S14

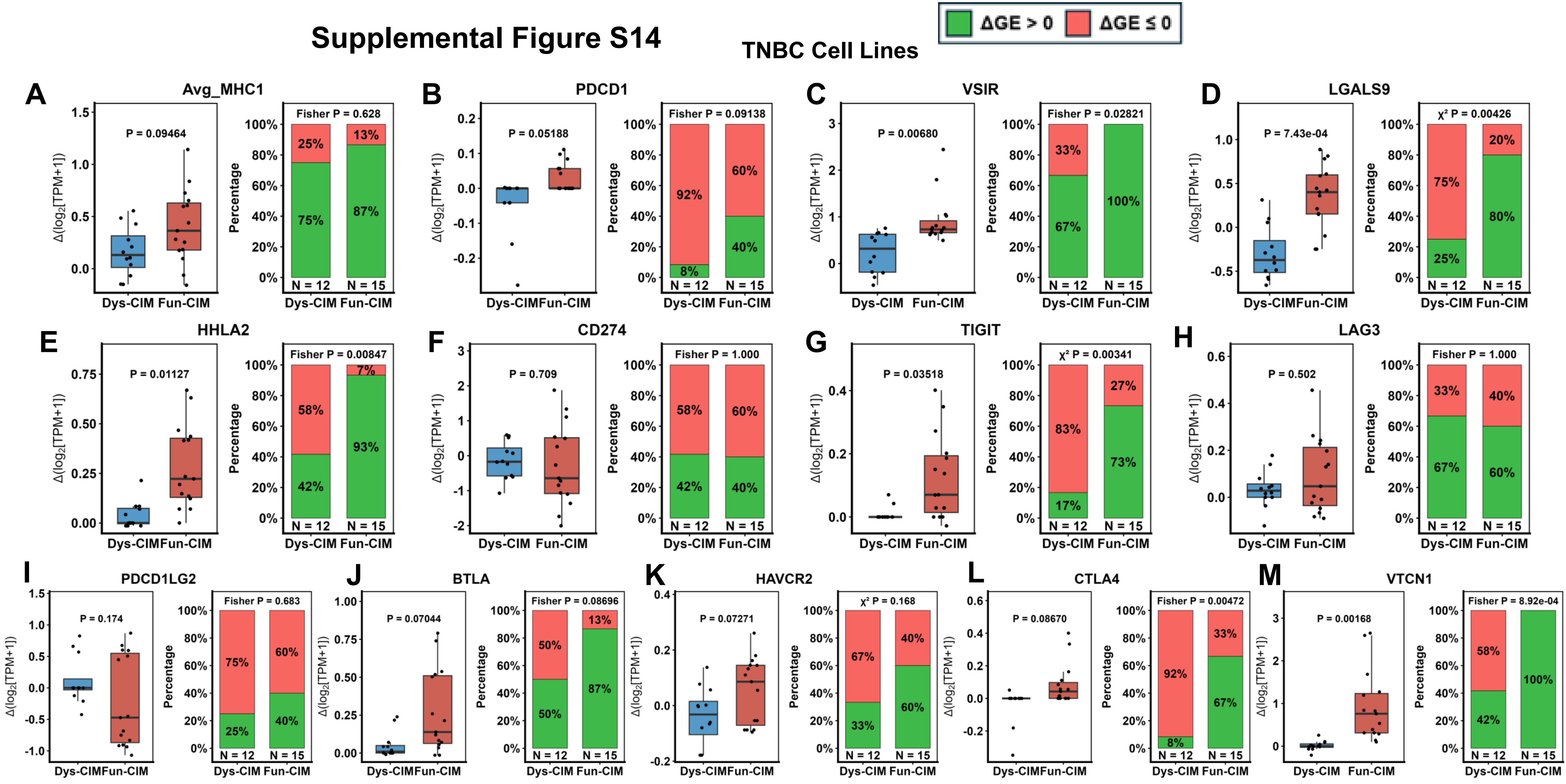

Supplemental Figure S15

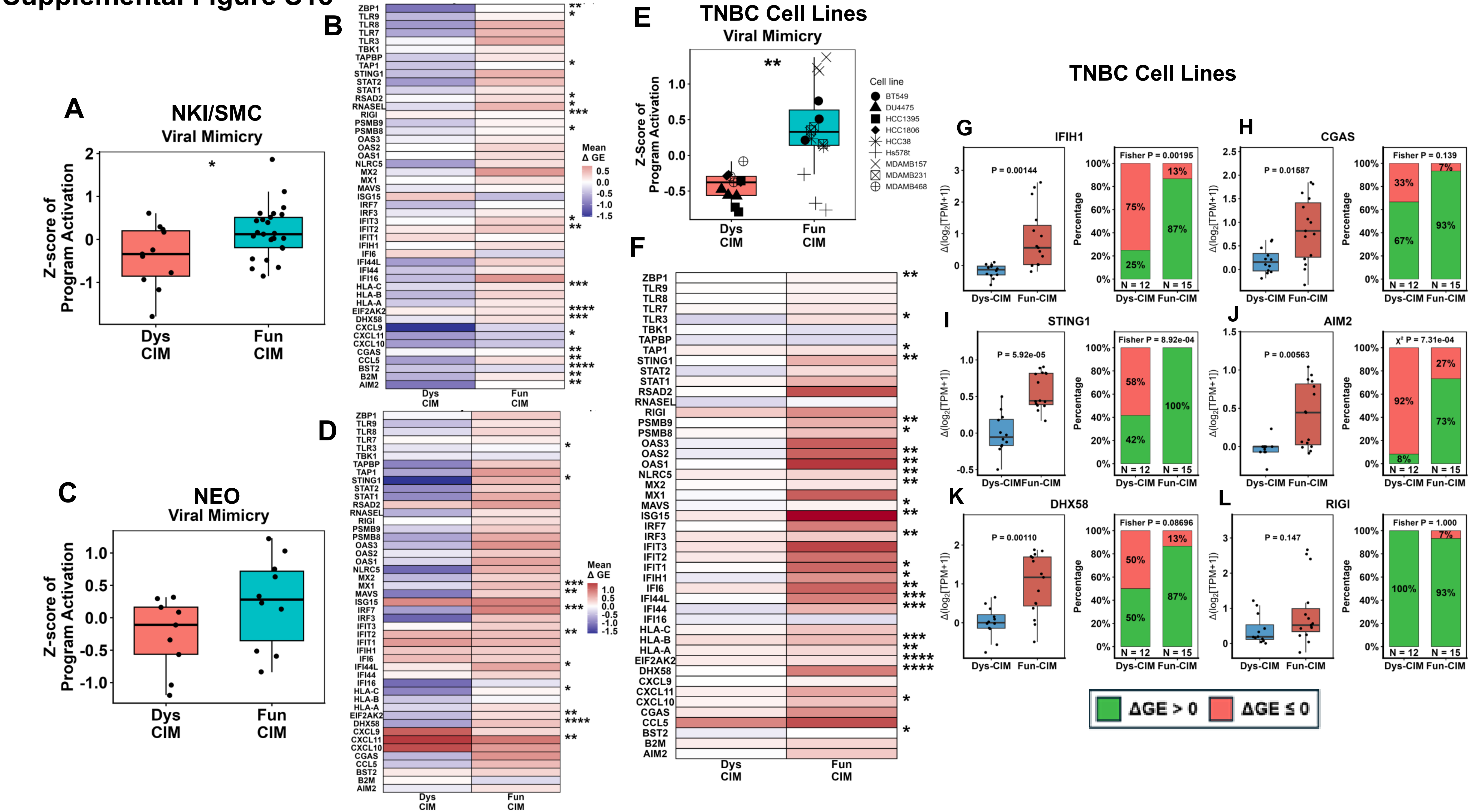

Supplemental Figure S16

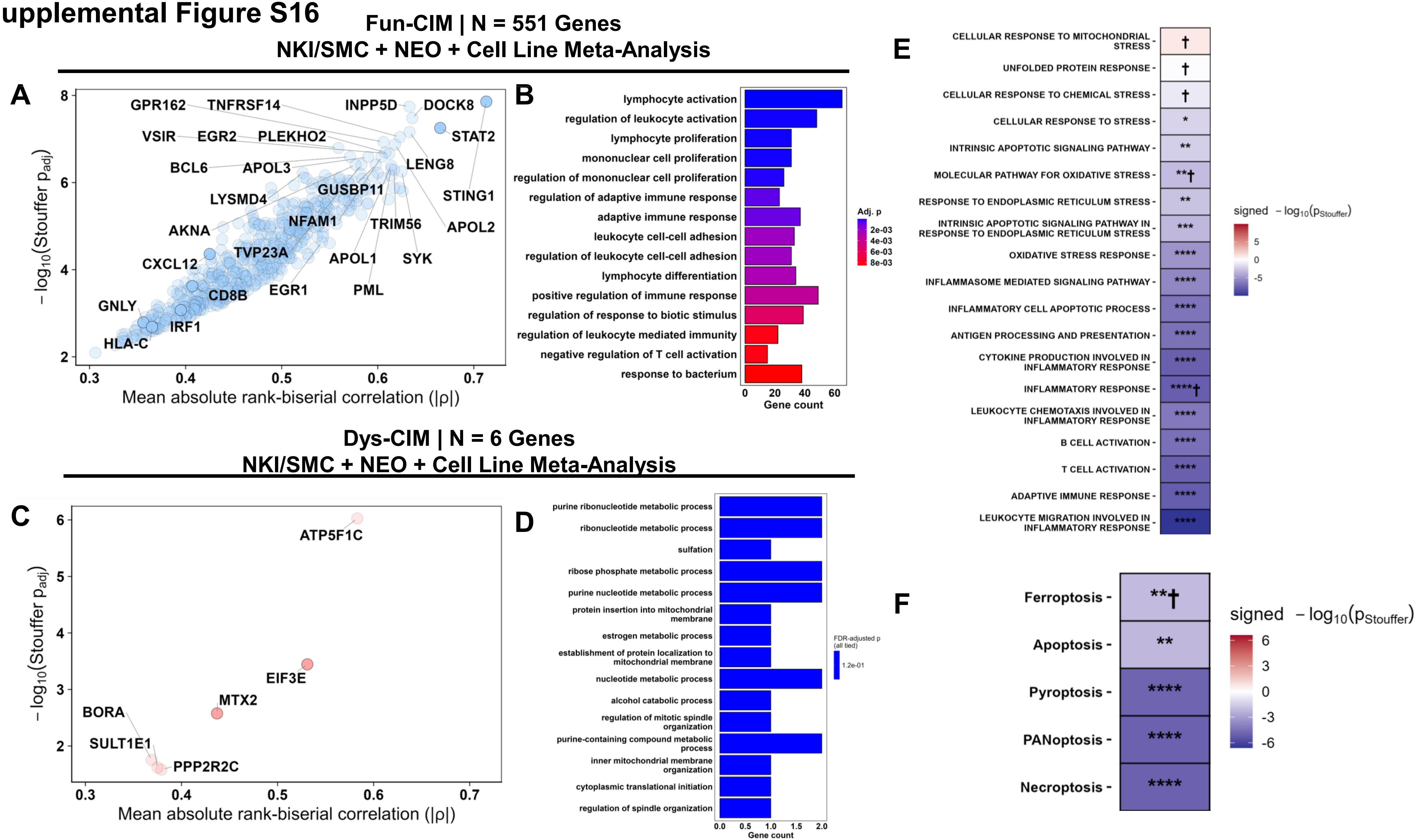

Supplemental Figure S17

NKI/SMC + Cell Line Meta-Analysis

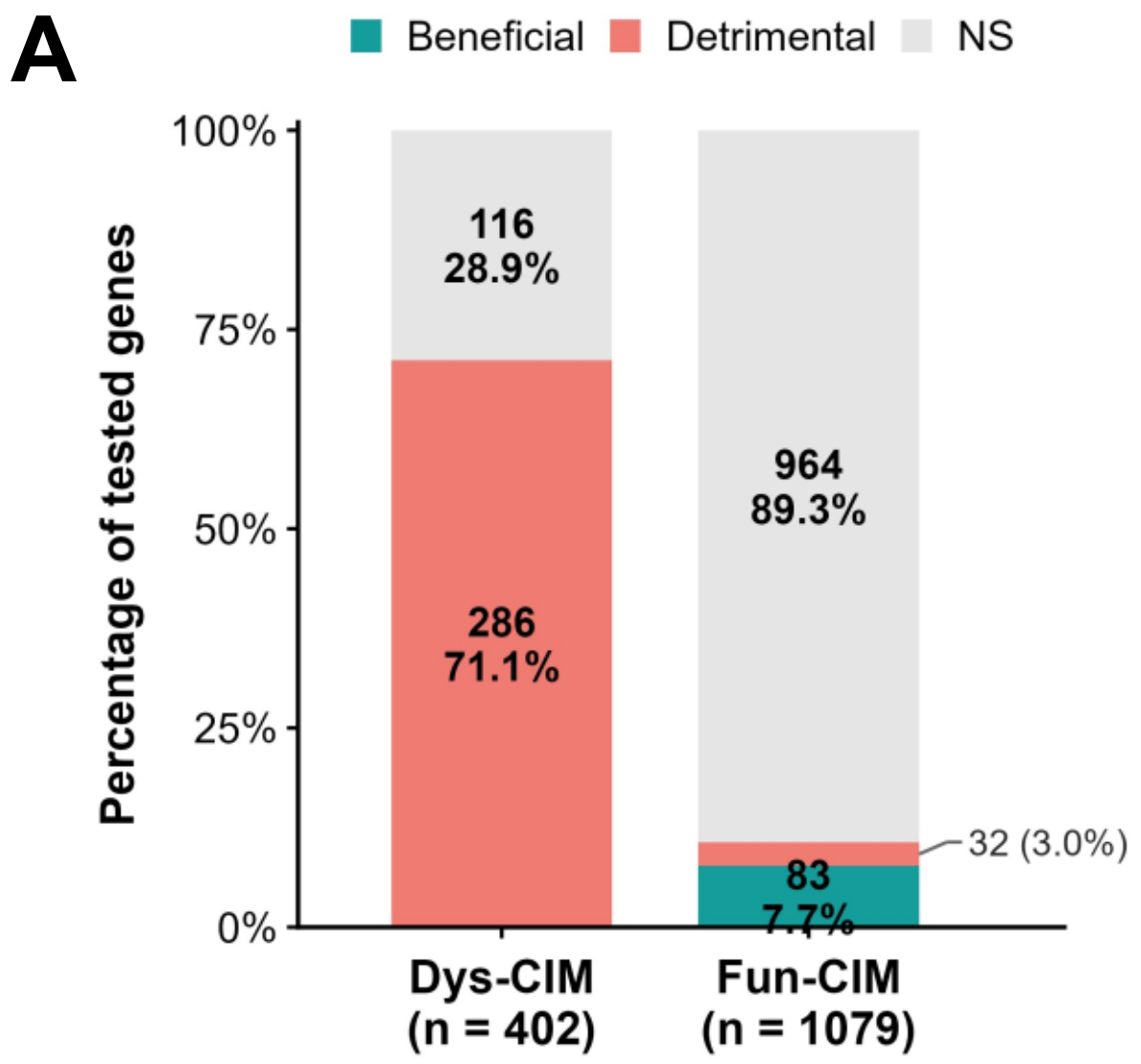

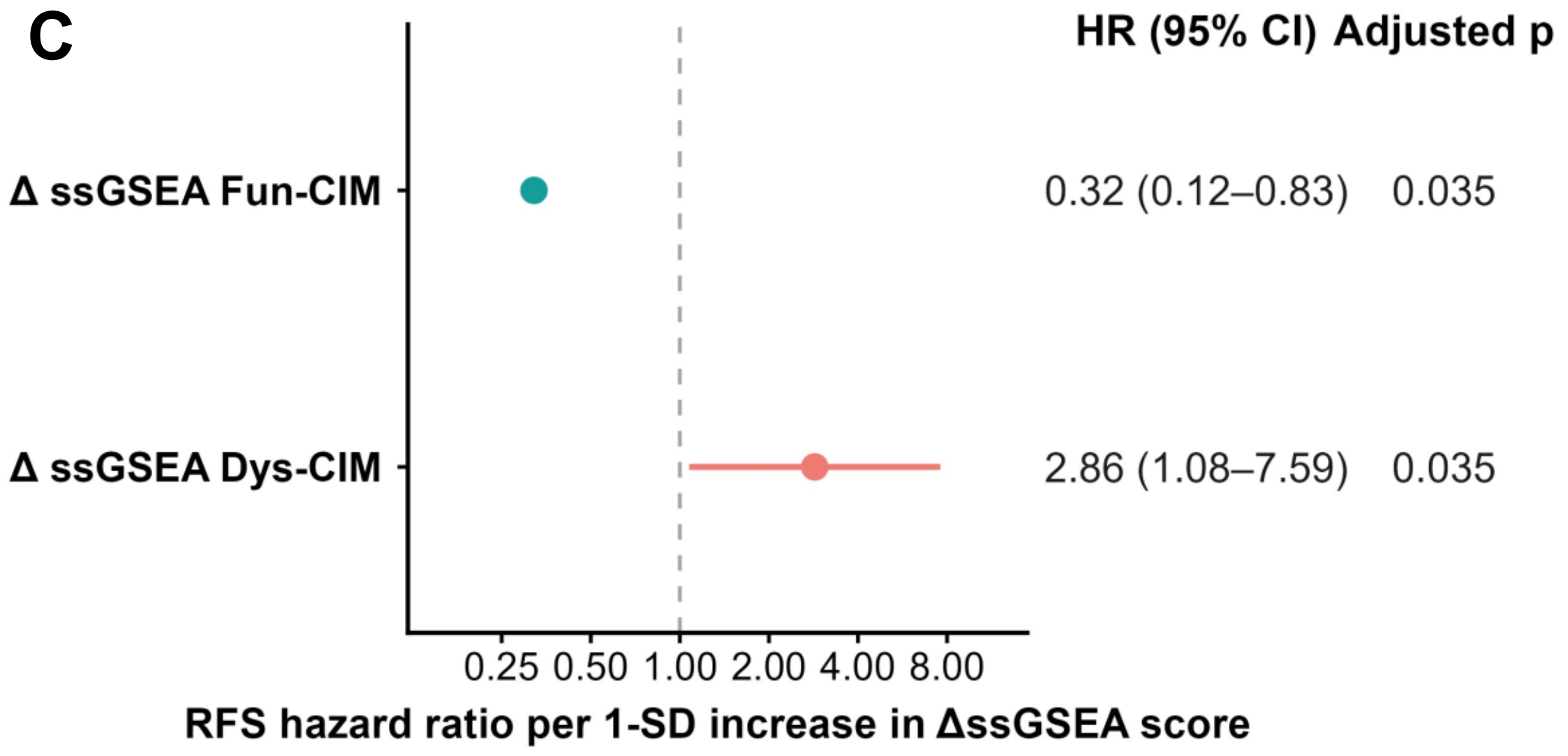

NKI/SMC + NEO + Cell Line Meta-Analysis

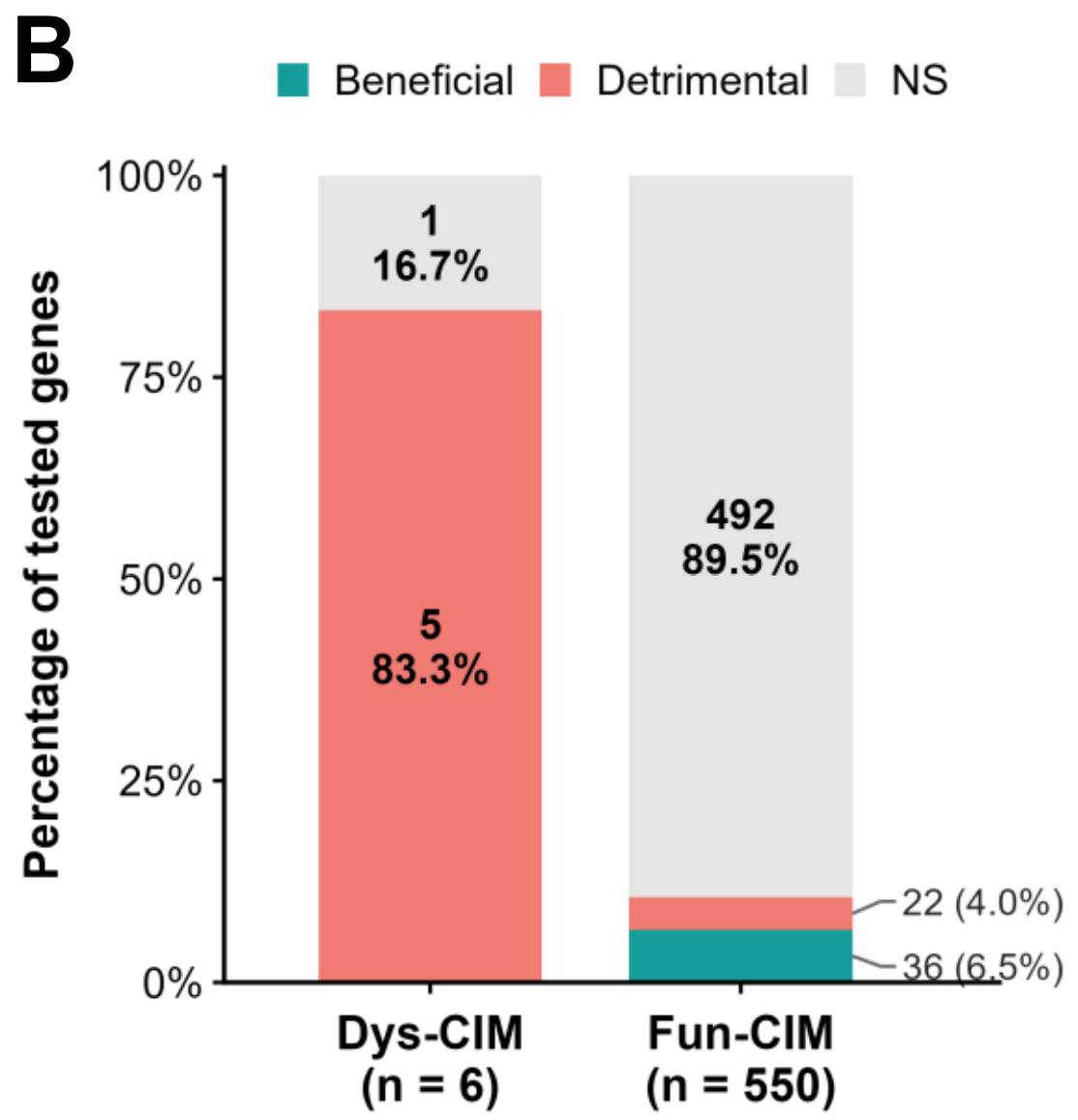

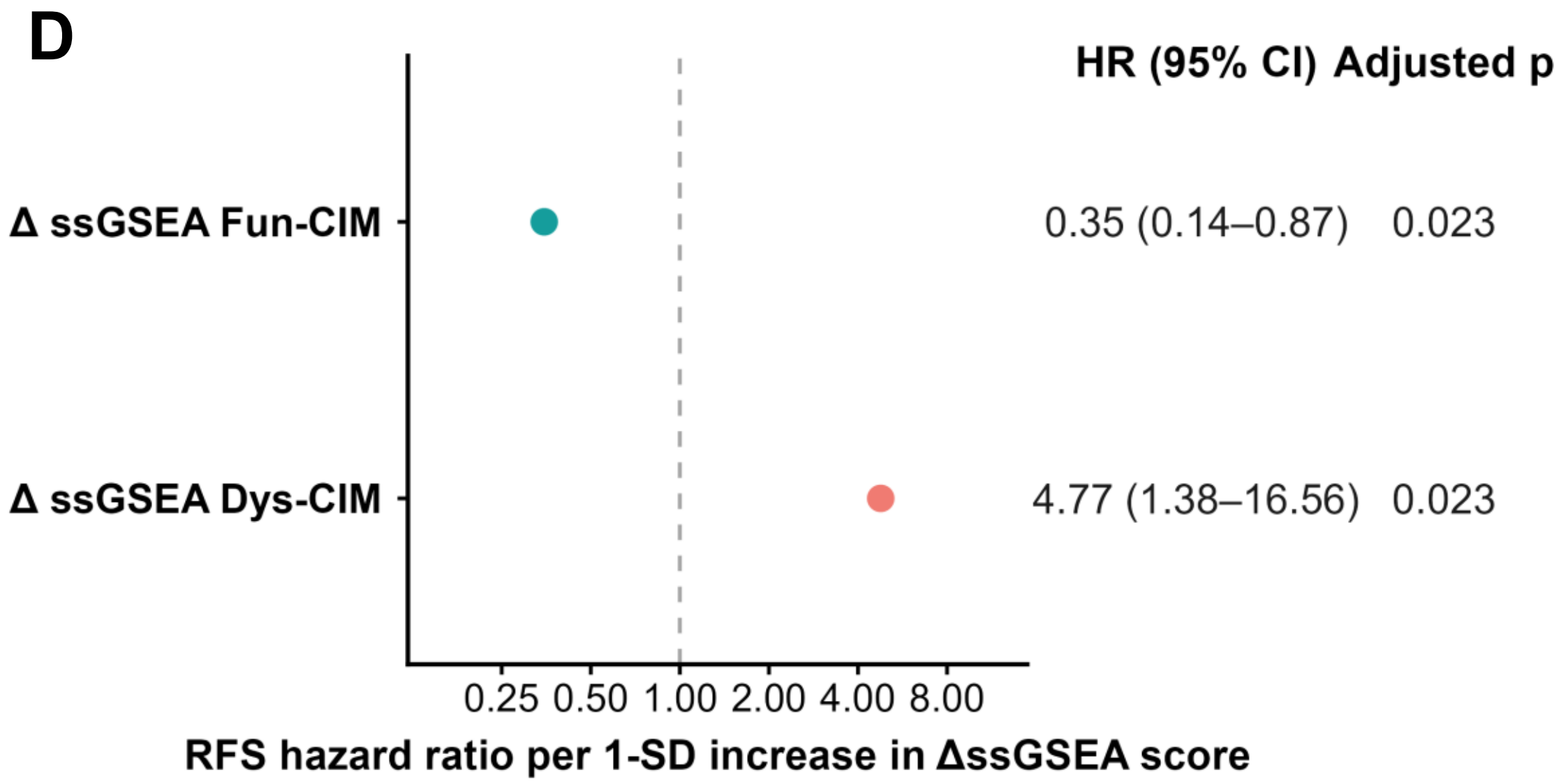

Supplemental Figure S18

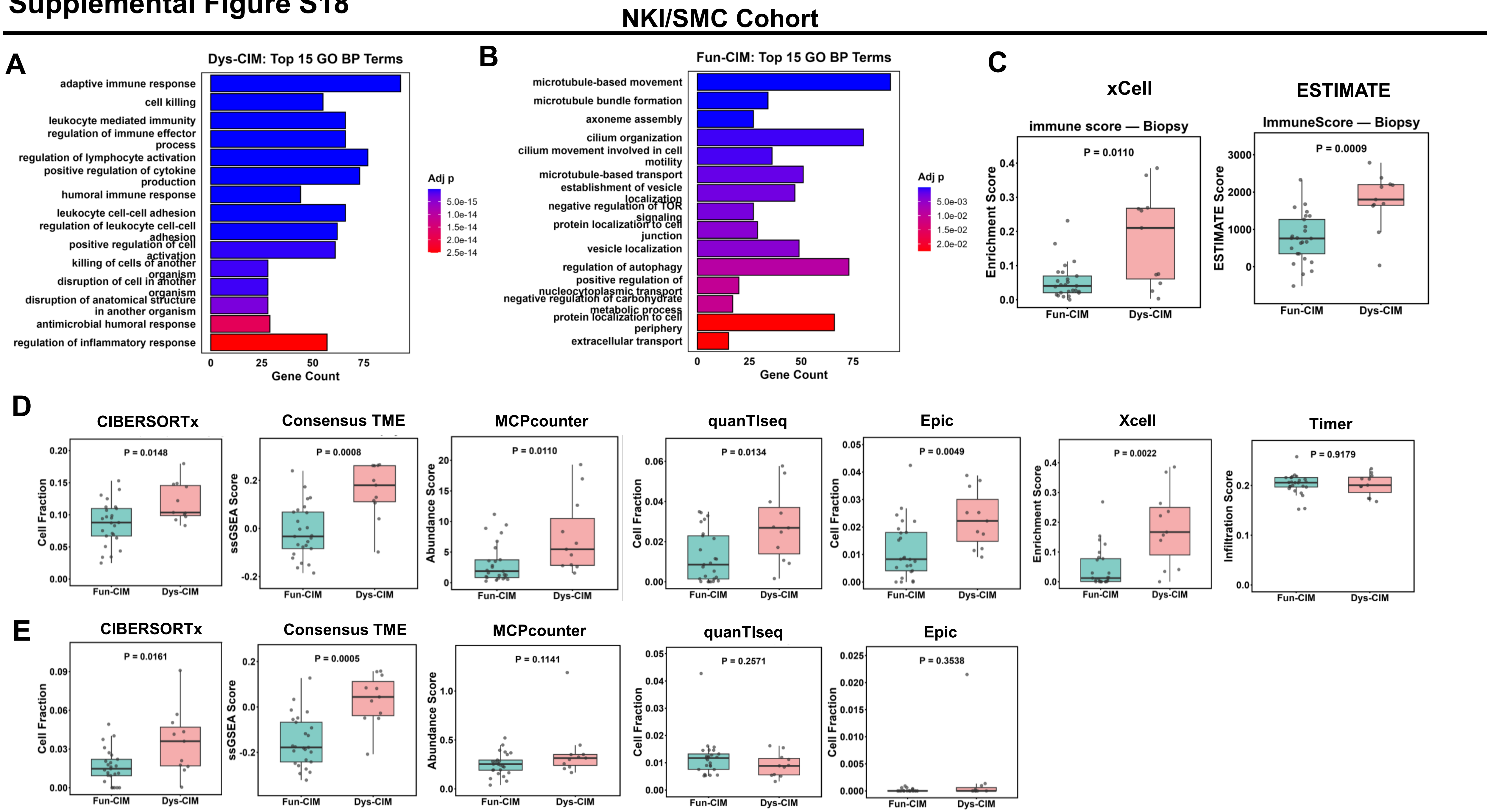

Supplemental Figure S19

NKI/SMC Cohort

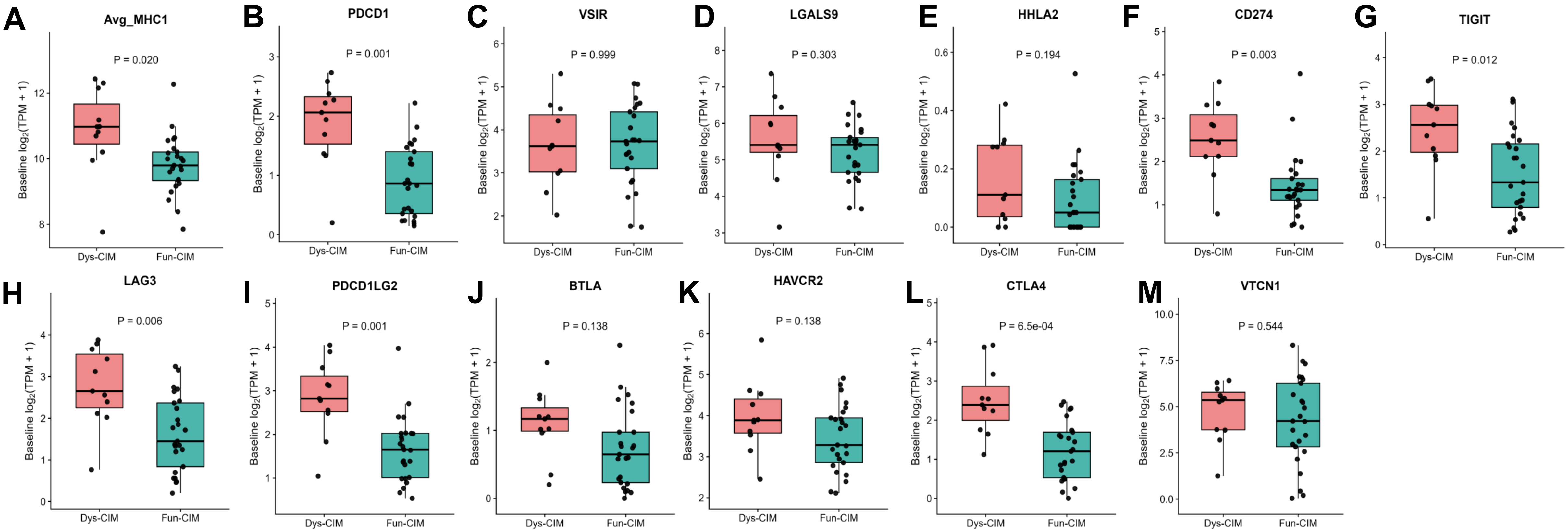

Supplement: Supplementary file 1 [file NIHPP2026.09.24.754178v1-supplement-2.pdf]
